# Protecting an island nation from extreme pandemic threats: Proof-of-concept around border closure as an intervention

**DOI:** 10.1371/journal.pone.0178732

**Published:** 2017-06-16

**Authors:** Matt Boyd, Michael G. Baker, Osman D. Mansoor, Giorgi Kvizhinadze, Nick Wilson

**Affiliations:** 1 Adapt Research Ltd, Wellington, New Zealand; 2 Department of Public Health, University of Otago, Wellington, New Zealand; 3 39 Mortimer Tce, Wellington, New Zealand; University of Michigan, UNITED STATES

## Abstract

**Background:**

Countries are well advised to prepare for future pandemic risks (e.g., pandemic influenza, novel emerging agents or synthetic bioweapons). These preparations do not typically include planning for complete border closure. Even though border closure may not be instituted in time, and can fail, there might still plausible chances of success for well organized island nations.

**Objective:**

To estimate costs and benefits of complete border closure in response to new pandemic threats, at an initial proof-of-concept level. New Zealand was used as a case-study for an island country.

**Methods:**

An Excel spreadsheet model was developed to estimate costs and benefits. Case-study specific epidemiological data was sourced from past influenza pandemics. Country-specific healthcare cost data, valuation of life, and lost tourism revenue were imputed (with lost trade also in scenario analyses).

**Results:**

For a new pandemic equivalent to the 1918 influenza pandemic (albeit with half the mortality rate, “Scenario A”), it was estimated that successful border closure for 26 weeks provided a net societal benefit (e.g., of NZ$11.0 billion, USD$7.3 billion). Even in the face of a complete end to trade, a net benefit was estimated for scenarios where the mortality rate was high (e.g., at 10 times the mortality impact of “Scenario A”, or 2.75% of the country’s population dying) giving a net benefit of NZ$54 billion (USD$36 billion). But for some other pandemic scenarios where trade ceased, border closure resulted in a net negative societal value (e.g., for “Scenario A” times three for 26 weeks of border closure–but not for only 12 weeks of closure when it would still be beneficial).

**Conclusions:**

This “proof-of-concept” work indicates that more detailed cost-benefit analysis of border closure in very severe pandemic situations for some island nations is probably warranted, as this course of action might sometimes be worthwhile from a societal perspective.

## Introduction

A widespread view is that country border closures have a limited, if any, role in preventing the spread of infectious diseases [1]. Indeed, the World Health Organization (WHO) advice is that even though unaffected countries may be able to delay the introduction of the infectious agent by imposing severe limits on international travel, border closure is unlikely to be able to prevent importation, and can have huge economic and personal costs. Such border closures can potentially damage trade and economies with one US study estimating the GDP halving from one year’s closure that suspended 95% of imports and exports [2].

Yet, historically there is evidence that border closure can be effective in preventing the spread of pandemic influenza: successful control of the 1918–19 influenza pandemic in various Pacific islands [3] and US military bases [4]. Internal border control was also helpful for Iceland in that pandemic [5]. Theoretical studies of small island nations also suggest that stopping travel in response to an influenza pandemic might be helpful in those with low travel volumes [6].

Furthermore, it is biologically plausible that a new infectious agent (e.g., a synthetic bioweapon or an existing agent like Ebola that became more infectious), could be of such severity that attempting border closure could be a rational choice for a country, particularly for island nations. That is especially if that closure decision could be made in time before the agent had already entered the island nation in sufficient numbers to prevent local control and that border controls could be successfully maintained for the period of risk.

Clearly, a new virus that had the infectiousness of measles or influenza and the lethality of rabies or Ebola would justify extraordinary control measures. Such a pandemic would cause severe economic impacts, with or without border closure, if the spread of the virus (or other pathogen) was not controllable. This situation is in contrast to the way that SARS, MERS-CoV and Ebola are relatively controllable since transmission does not usually occur until after severe symptom onset.

So, can we reframe the question to defining the severity of a new pandemic that would justify consideration of border closure, for countries where this might be feasible e.g., by an island nation? This study aims to explore that question at in initial “proof-of-concept” level using the island nation of New Zealand as a case study.

New Zealand has the benefits of being geographically remote (so that alternatives to aircraft travel are difficult) and it has a very well developed border control system, which is designed particularly to exclude pathogens that threaten agriculture (such as foot and mouth disease). New Zealand is also relatively well organized when it comes to disease control in that it has successfully eradicated brucellosis, hydatids and more recently an invasive mosquito species that had spread to multiple parts of the country [7]. On the other hand, New Zealand is a nation that is highly dependent on tourism revenues and on exports–so these are components of the economic harm that border closure can cause to a country’s economy.

## Material and methods

Our approach was of an initial proof-of-concept level analysis to estimate costs and benefits of border closure using Excel spreadsheet modeling. A societal perspective was adopted and costs and benefits were discounted at 3%. The model extrapolated existing data on past influenza pandemics in New Zealand to calculate the number of: primary care visits, hospitalizations, and deaths predicted for various pandemic scenarios. These scenarios varied by the proportion of the population infected, being admitted to hospital and dying. The estimated cost of the pandemic to the country was calculated under a range of assumptions, including monetizing quality-adjusted life-years (QALYs) and deaths, accounting for lost tourism revenue and export/import losses. Scenarios that account for health system only costs are also reported. The model can be accessed in S1 Appendix. Details around the scenarios and parameters follow, with Table 1 listing all the epidemiological and costing input parameters used in the model.

**Table 1:** Epidemiological and costing input parameters used in the pandemic and border closure model as applied to the island nation of New Zealand (NZ).

Variable	Expected value	Distribution*	Data source/s and details
***Key epidemiological characteristics***			
Proportion (of the population) infected	Scenario A: 0.400	Beta (14.6, 21.9)	Wilson et al 2012 [9] (within the range of 33% to 50% for 1918 influenza pandemic data).
	Scenario B: 0.500		Capped at 50%.
Proportion of the population who consult their general practitioner (GP)	Scenario A: 0.059	Beta (23.5, 374)	Wilson et al 2012 [9] (2009 pandemic data).
	Scenario B: 0.100		Proportional to infected, capped at 10%.
Proportion of the population hospitalized	Scenario A: 0.0275	Beta (24.3, 859)	Wilson et al 2012 [9] (2009 pandemic data).
	Scenario B: 0.0275		Capped at same level as Scenario A.
Proportion of the population dying†	Scenario A: 0.00275	Beta (24.9, 9040)	Wilson et al 2012 [9] (1918 pandemic data)
	Scenario B: 0.0275		Capped at Scenario A x10
***Values used in estimating morbidity impacts (included in quality-adjusted life-year [QALY] calculations)***			
Disability weight (DW) for mild disease (1 week, no GP visit, not hospitalized)	0.0001	Beta (0.0017, 17.2)	Salomon et al 2012 [11]
DW for moderate disease (2 weeks, GP visit, not hospitalized)	0.0020	Beta (0.026, 12.5)	Salomon et al 2012 [11]
DW for severe disease (3 weeks, hospitalized)	0.0121	Beta (0.076, 3.31)	Salomon et al 2012 [11]
***Key cost-related parameters***			
Accommodation costs for international visitors stranded (per day)	NZ$200	Gamma (100, 2)	Authors’ best estimate.
Repatriation costs (airfare to Australia per visitor and/or air force flights)	NZ$1000	Gamma (100, 10)	Authors’ best estimate.
Average number of international visitors per day to NZ (number of visitor days per annum divided by 365)	151,251	Gamma (11.1, 13613)	NZ Ministry for Business, Innovation and Employment [16]
Value of saving a life in each five year age group (males and females combined)	E.g.: NZ$1,093,000 (for a 25–29 year old)	Normal	Kvizhinadze et al 2015 [12] (and online calculator) [13] (with NZ for country selection and taking into account age-specific background health costs)
Cost per GP visit	NZ$60	Gamma (100, 0.6)	NZ Ministry of Health Subsidy for screening visits
Cost per hospitalization	NZ$20,225	Gamma (48.3, 418.5)	Wilson et al 2012 [8] (2009 influenza pandemic data)
Average revenue per visitor to NZ per day (total visitor spend)	NZ$125	Gamma (100, 1.25)	NZ Ministry for Business, Innovation and Employment [16]
***Supplementary analyses***			
Cargo-based Imports	NZ$961 million [m] per week	Point estimate	Statistics New Zealand published economic data [17]
Cargo-based exports	NZ$999 m per week	Point estimate	Statistics NZ published data [18]

* Distributions given for Scenario A only, shape and scale parameters given in brackets.

† The derived case fatality rates are therefore: Scenario A = 0.69%, Scenario B = 5.5%

### Base-case scenarios

We considered novel pandemic scenarios that would typically involve infectious respiratory agents spread between people and causing rapidly propagating acute illnesses. These could conceivably arise from natural phenomena such as new influenza pandemics, but also from the unintentional or purposeful release of a modified pathogen or bioweapon with respiratory spread. Such synthetic agents could conceivably have the infectivity of pandemic influenza and the mortality risk of viral hemorrhagic fevers.

Nevertheless, the key aspects of the pandemic scenarios were scaled from historical experience with influenza pandemics in the New Zealand setting. Scenario A was a serious pandemic that was assumed to have similar morbidity impacts to the 1918/1919 influenza pandemic, but with half the mortality rate–given modern improvements in supportive health care and treatments (Table 1). Scenario B was of a particularly extreme pandemic that had ten times the mortality impact as “Scenario A”.

The border closure intervention was assumed to completely prevent any harm to health from the threat of these two pandemic scenarios. Border closure was assumed to include an end to the arrival of all commercial passenger planes, private planes, cruise ships, and yachts. If trans-oceanic yachts or private jets attempted to enter territorial space after closure, it was anticipated that they would be detected and managed by New Zealand’s military (e.g., forced to go to other countries or to uninhabited off-shore islands around the coast).

Our base-case analysis was focused on the impact of reduced tourism from border closure as we assumed that trade in exports and imports would continue with appropriate controls to ensure complete isolation of incoming air cargo crews and ship crews in special port facilities or with ship crews never leaving their vessels. Indeed, the latter is already sometimes the case when ships load/off-load at New Zealand ports.

### Pandemic epidemiology

The proportion of the population infected was taken from influenza pandemic data for New Zealand in 2009 [8] and 1918 influenza pandemic mortality data for New Zealand (Table 1). The proportion of the population consulting their general practitioner (GP) and the proportion hospitalized were taken from the literature for the 2009 influenza pandemic in New Zealand [9]. The ratio of the proportion infected in the 1918 pandemic to the proportion infected in 2009 allowed us to estimate the number likely to consult their GP in the Scenario A pandemic (by holding the ratio of GP 1918:GP 2009 consistent with the ratio of proportions infected). In the more extreme Scenario B pandemic, we assumed 50% of the population would be infected, 10% would consult their GP and 10 times as many would die as in the Scenario A pandemic.

Age-specific patterns of hospitalizations and deaths were taken from published New Zealand data on the 2009 influenza pandemic [10] and applied to all scenarios. Population data by five-year age groups in 2014 were used for calculations.

### Health impact parameters

For the Scenario A pandemic, we halved the mortality rate relative to that from the 1918 influenza pandemic. This meant that instead of the estimated European New Zealanders mortality rate of 550 per 100,000 population we used a mortality rate of 275 per 100,000 population (Table 1). This was based on the assumption that provision of basic supportive care would be better than for the 1918 pandemic and so would the provision of modern treatments before health services got overloaded (e.g., potentially some people could benefit from modern treatments such as antibiotics to treat bacterial pneumonia that followed the pandemic infection). Deaths in the more extreme Scenario B pandemic were modeled as 10 times greater than the 1918 scenario (Table 1). We assumed ‘W-shaped’ mortality rate distributions with peak death rates for children and younger adults, as was the case for the 2009 and 1918 influenza pandemics.

The health burden included assessing QALYs lost from different health states. That is, there was health loss for those infected in the pandemic, those consulting their GP, those who were hospitalized, and those dying prematurely. Calculating the non-fatal health impact involved allocating a weighted annualized acute infectious disease disability weight (DW; mild, moderate, severe respectively) according to those from the Global Burden of Disease Study [11]. Infected people were modeled as experiencing one week with the DW, those consulting their GP experienced two weeks, and those hospitalized received three weeks. The DWs were multiplied by (1-pYLD [prevalent years lived with disability]), to account for the proportion of years lived with disability derived from New Zealand life-tables.

### Costs

A large driver of the costing part of the analysis was the estimated value of saving a life at different ages and for this we used a tool which calculates the “Expected Maximum Intervention Cost (EMIC)” to save a life for different countries [12]. More specifically, this is the amount of money that a society might spend on a one-off intervention to save the life of an individual given their age, while accounting for life-time health costs and allowing for country-specific values for a QALY gained. The online calculator allows for country-specific estimates of these values, but we used the values for New Zealand in the main analysis [13]. For the cost-effectiveness threshold in the equation used, we applied the rule-of-thumb approach proposed by the WHO: those interventions with a cost per QALY gained that is less than the country’s GDP per capita can be considered very cost-beneficial [14]. For this analysis, that equates to a relatively conservative cost-effectiveness threshold of NZ$45,000 per QALY gained (for year 2011; $US30,000 adjusted for OECD purchasing power parity [15]). QALYs gained from morbidity reduction were also monetized at this same level.

We used a discount rate of 3% per annum when accounting for the future health system costs and valuation of QALYs gained by implementing border closure. At this discount rate the value of saving a life for New Zealand citizens in the age ranges 0–4, 35–39, and 65–69 are: NZ$1.288 million, NZ$994,000, and NZ$521,000 respectively.

In terms of direct health care costs, GP visit costs were set at NZ$60 per visit. Hospitalization costs were those that have previously been estimated for the 2009 pandemic in New Zealand [8].

The New Zealand Ministry for Business Innovation and Enterprise publishes annual international visitor numbers and revenue [16]. From this we could calculate the average number of international visitors in New Zealand on the day of border closure and the lost revenue in terms of daily visitor spending (by different periods of border closure). In some scenarios we also included the costs of government-funded provision of accommodation for the tourists remaining in New Zealand (estimated at NZ$200 per day per person), and the costs of evacuating visitors by essentially “one-way” flights home (estimated at NZ$1000 per individual).

Border closure enforcement costs (including guarding cargo ships) were ignored due to the complexity of this aspect but also because they may be relatively low compared with the normal routine costs to customs agencies processing thousands of arrivals per day, which would cease during the border closure period. Similarly, existing military personnel could be diverted from their base camps to assist with such tasks. We also ignored the costs of using air force and naval patrols to ensure border protection (e.g., from arriving trans-oceanic yachts) as these type of patrols are already conducted (for fisheries protection).

### Statistical and uncertainty analysis

An expected value for each input variable was calculated and a distribution estimated. We ran a probabilistic sensitivity analysis (PSA) on the base case using values sampled randomly from the distributions for all variables and 1000 iterations. For this the Excel add-in tool Ersatz (EpiGear, Version 1.3) was used. The cost-benefit results for other scenarios were calculated using expected values for the variables.

### Additional scenarios

We explored two additional scenarios in which import/export trade: (i) was reduced by 50%; and (ii) ceases completely:

1. All cargo imports and exports in and out of New Zealand were assumed to be via one highly-guarded shipping port and one highly-guarded airport with these facilities also having protective sequestration for port workers as an extra precaution. As a result, we assumed that this would constrain the total value of cargo-based exports and imports by 50% during the “border closure” period. Also assumed was a recovery period of three months after the border was reopened until normal cargo trade for exports and imports was resumed (assuming a linear increase). We used 2014 data for the value of cargo-based imports at NZ$961 million per week [17] and of cargo-based exports at NZ$999 million per week [18]. When annualized, these amounts comprise 20.7% and 21.6% of New Zealand’s GDP respectively (which was NZ$241.2 billion in the year ending March 2015 [19].
2. In this extreme scenario we assumed a complete end to all cargo imports and exports in and out of New Zealand during the “border closure” period. This would effectively double the impact of the scenario detailed above. Similarly, we also assumed a recovery period of three months after the border was reopened until normal cargo trade for exports and imports was resumed (assuming a linear increase).

## Results

The summed costs associated with no border closure are listed in the first row of Table 2. These include discounted monetized QALYs lost from illness and premature death, and also discounted healthcare costs associated with the pandemic. These costs were estimated to range from NZ$14.4 billion (US$9.63 billion) for the Scenario A pandemic, where the country suffers 12,400 pandemic-related deaths, to NZ$121 billion for the Scenario B pandemic, in which the country suffers 124,000 deaths.

**Table 2:** The net benefit over cost to New Zealand (NZ$ billions) under various pandemic scenarios when compared with the expected cost of the pandemic given no border closure (positive values reflect net societal benefit, negative values (in bold) reflect net societal loss).

Scenario details and variants		Scenario A (NZ$ billions)	Scenario B (NZ$ billions)
Cost of pandemic with no border closure		14.4	120.6
***Assuming closure prevents 100% of cases (value given is net benefit to the whole of society)***			
1. Base border closure scenario	26 weeks closure	$11.0	$117.2
	12 weeks closure	$12.8	$119.0
2. Border closure with 26 week linear return to normal visitors	26 weeks closure	$9.3	$115.5
	12 weeks closure	$11.1	$117.3
3. Border closure with 26 week linear return after an initial 50% tourist downturn, evacuation costs, and 2 weeks accommodation costs	26 weeks closure	$11.3	$117.5
	12 weeks closure	$12.2	$118.4
***Scenario with failed border closure***			
4. Border closure with 26 week linear return to normal visitors (as above) but closure fails and the net result is a pandemic with 10% fewer cases/deaths	26 weeks closure	**-$3.7**	$6.9
	12 weeks closure	**-$1.9**	$8.8
***Without monetizing QALYs associated with premature death and illness***			
5. Border closure accounting just for direct health system costs of pandemic, 26-week linear return after an initial 50% tourist downturn, evacuation costs, and 2 weeks accommodation.	26 weeks closure	**-$0.6**	**-$0.6**
	12 weeks closure	$0.3	$0.3

Where border closure was assumed to prevent the Scenario A pandemic, it was estimated to provide a large net societal benefit (is very cost-beneficial) for both 12 and 26 weeks of closure (Table 2). For example, if closure was 12-weeks duration, with a 26-week linear return to normal tourist visitor numbers, then successful closure was estimated to save NZ$11 billion when compared to no closure. Even more clearly, border closure for Scenario B was found to be highly cost-beneficial under all assumptions, saving up to NZ$120 billion to society (Table 2).

If border closure failed, and as a result the pandemic reached New Zealand (albeit with the assumption of 10% fewer cases/deaths due to benefits from some delay), then closure would no longer be cost-beneficial in Scenario A (Table 2). However, at this modest level of health benefit, failure of closure in Scenario B would still return a net societal benefit of at least NZ$7 billion.

For border closures to prevent pandemic Scenarios A and B, the cost-benefit analysis results were largely driven by the valuation of potential premature deaths averted (the lost life-years component of the QALYs). Nevertheless, when we removed the valuation of life and the monetization of these lost QALYs from the calculations, a 12-week border closure was still cost-beneficial for some scenarios. For example, for the Scenario A pandemic with a subsequent 50% tourism downturn, 26-weeks linear return to normal visitor numbers, and visitors’ accommodation and flights home subsidized by the government, closure was still estimated to be beneficial overall by NZ$300 million compared with no closure. The benefit was very similar for Scenario B because we capped the number of hospitalizations, given that the health system was assumed to be unlikely to cope with the high numbers in this scenario.

Various threshold effects can be determined from these results. For example, a net societal benefit was estimated for closing the borders for 50 weeks if 0.15% of the population were predicted to die in a pandemic and if border closure was successful (Figure A in S2 Appendix).

The results of the additional scenarios are presented in Table 3. That is, if disruption of trade (imports and exports) was also taken into account in the Scenario A pandemic, it was not estimated to be economic to close the borders for either 12 or 26 weeks. But for the Scenario B pandemic it remained very worthwhile to close the borders, even when taking into account export and import losses of 100%.

**Table 3:** Additional analyses when considering the net benefit to New Zealand of border closure when allowing for reductions or elimination of imports and exports during the border closure period (net societal loss is indicated in bold).

Scenario*† (export + import % reduction)	12 weeks border closure (+12 weeks linear return) (NZ$ billions)	26 weeks closure (+12 weeks linear return) (NZ$ billions)
1. Scenario A (50%)	**-5.6**	**-21.2**
2. Scenario A (100%)	**-23.2**	**-52.5**
3. Scenario B (50%)	+100.6	+85.0
4. Scenario B (100%)	+82.9	+53.7
5. Scenario A x 5 (100%)‡	+23.9	**-5.3**
6. Scenario A x 3 (100%)‡	+0.4	**-28.9**

* These use the ‘base scenario’ i.e. we don’t account for costs of returning visitors home, nor do we account for their accommodation costs.

† An example of how the calculation works, in this instance it is the sum of: (Costs averted (hospital and GP costs + premature deaths (monetized QALYs) + morbidity (monetized QALYs) = $120.6 billion [b]) MINUS (Tourism losses = $2.4b) MINUS (Import + Export losses = $35.3b) = $82.9b

‡ Of note is that pInfected is capped at 0.5, pGP is capped at 0.1, pHospitalized is capped at 0.0275, but pDeath is allowed to vary e.g., by x3, x5, of Scenario A pandemic.

For a pandemic with five times the adverse mortality impact of Scenario A, there was a period of closure between 12 and 26 weeks at which the net benefit switched from favorable to unfavorable (Table 3). For a situation where the pandemic had three times the adverse mortality impact of Scenario A, it was found to be marginally net beneficial to close the border for 12 weeks (and account for 100% export/import losses) but any longer than 12 weeks and there was a net cost to society.

### Sensitivity analyses around the base-case

We ran a probabilistic sensitivity analysis (PSA) using distributions for all variables and 1000 iterations. The proportion dying from the pandemic was the most influential parameter on the net economic impact of the base-case for Scenario A, resulting in societal savings varying from $7.3 to $16.8 billion (Tornado Plot in Figure B in S2 Appendix). Less important variables in driving the uncertainty were visitors per day, duration of closure, and the value of saving a life estimates (Figure B and Figure C in S2 Appendix). When QALYs were not monetized, the proportion being hospitalized was also an important contributor to the uncertainty (Figure C in S2 Appendix).

## Discussion

Human civilization is at ongoing risk of new infectious agents. This risk has probably never been higher because of growing human population density and technology leading to socio-economic, environmental and ecological factors [20]. There are also added threats from deliberate human modification of existing or potential pathogens [21]. Consequently, it seems appropriate that governments should prepare their societies for dealing with global pandemic threats.

This initial “proof-of-concept” work suggests that there are plausible conditions under which it appears cost-beneficial at the societal level to close the borders of an island nation in the face of a new and very severe global pandemic. A key assumption is some chance of closure being successful. The main variable dictating the threshold for achieving net societal gain appears to be the expected number of deaths from the pandemic and hence the monetized value of QALYs gained from border closure.

The severity threshold where border closure is likely to be justified is (not surprisingly) higher in situations where the duration of closure needs to be prolonged and/or the effects on export and import losses are also considered. Therefore, any comprehensive pandemic plan by an island country could include ways of reducing these negative effects (e.g., by planning contingency measures to support continuing export and import activities during such periods).

### Strengths and limitations of this study

A strength of this study is that it appears to be the first to attempt to weigh the economic and health issues associated with pandemic-related border closure at an island country level. It benefited from detailed epidemiological and costing data from past influenza pandemics for New Zealand, and also a published tool for estimating the value of saving a life at different ages [12]. Nevertheless, specific limitations are as follows:

- The method used monetized QALYs (based on GDP per capita valuation as used in WHO CHOICE work) which although having an element of resource realism, is still somewhat arbitrary. Also some societies may use different discount rates than the 3% that was applied to both costs and health gains in this study.
- The method did not explicitly include productivity benefits (in workers and potential workers whose lives are saved), although this benefit can be considered to be partly captured in the monetized QALY.
- In terms of the scenario analyses around the end to imports and exports, this analysis also did not consider a range of other relevant issues. That is for some export industries delays of some months might only result in partial economic losses. In New Zealand’s case, trees used for export timber can be left growing, beef cattle can be left on pasture, processed timber, wool and dried milk powder can be stockpiled, as can the refrigerated goods that the country exports (e.g., butter, cheese, meat products, onions and apples). Alternatively, some of these designated exports could be diverted to the domestic market, resulting in cheaper food for New Zealand citizens. Similar arguments might also apply to imports with some of these goods able to be stockpiled in foreign ports until the New Zealand border closure ended. Furthermore, some imports could be replaced by New Zealand-produced products. For example, food imports are largely discretionary for New Zealand at present given the country is a major producer (and exporter) in all of these major food categories: fruit, vegetables, dairy, meat and fish.
- Similarly, the valuation of lost tourism revenue could have been more sophisticated if a more detailed analysis considered the marginal cost of providing the tourism services being subtracted from the revenue gained. On the other hand, the estimates under-value the losses given that there is also no accounting for consumer surplus in the tourism experience for international visitors (though this might be less of a concern from a New Zealand Government perspective).

### Possible implications for further research

Our work is of an initial proof-of-concept nature relating to a non-specific “severe pandemic” threat. The results suggest that for this case study of an island nation, there are plausible pandemic conditions in which border closure might sometimes be worthwhile at a societal level. What may be needed next is further clarification of the precise nature of those conditions. We have not taken into account many costs that will be incurred and some that will be averted in a border closure situation. It is now up to specialist policy institutions such as economic ministries and treasuries to further investigate this line of reasoning in island nations with pandemic plans. This consideration should ideally involve more sophisticated modeling of many important parameters and also include updated uncertainty analysis. There may also be a place for multiple-criteria decision analysis (MCDA) that would allow consideration of wider factors e.g., if a new synthetic bioweapon posed existential risks to a society, then possibly very rapid and extreme border controls might be justified in an island nation. For less severe pandemics, a MCDA approach may also capture considerations around more adverse health and economic impacts on low-income populations that could exacerbate inequities, and other ethical complexities (e.g., not allowing citizens overseas to return).

### Possible implications for policy in island nations

While further research is necessary, these initial modeling results suggest that border closure could reasonably be on the menu of pandemic planning options for island nations. Furthermore, even if border closure fails to prevent the pandemic’s arrival in the threatened island nation, a period of border closure could delay the arrival of the pandemic (given evidence from border screening activities for the 2009 influenza pandemic) [22]. Indeed, even short border closures could buy time to allow for more detailed epidemiological assessment, time to launch a national mass media campaign around hygiene and staying at home if sick, time to prepare healthcare facilities, and time to vaccinate key front line staff.

These results also suggest the potential utility of a forecasting system that could be used in real-time at the early stages of a new pandemic with high mortality potential. A workable policy would need the capacity for rapid border closure before detailed knowledge about the nature of the novel pandemic agent. However, this level of timeliness could be problematic as shown by the difficulty detecting pandemic influenza during its early establishment stages (e.g., as observed in parts of Australia in 2009 [23]). Indeed, even well-organized governments in island nations should assume a relatively high possibility that border control might not be enacted in time or might subsequently fail.

Any provisional border closure plans should probably include consideration around facilities that allow for very effective protective sequestration of in-coming ship or cargo airline crews (e.g., of airline crew that need to sleep before flying out). Rigorous precautions are potentially very important given that if closure fails to prevent 100% of cases in a Scenario A type pandemic, and instead 90% of the expected cases actually occur, then the cost-benefit calculation can flip (e.g., by NZ$10–12 billion in this case study from being massively cost-beneficial, to a substantial net societal loss).

Finally, the topic of border closure for different types of countries also needs to be further refined at the international level so that the World Health Organization can provide the best available guidance on the topic. The International Health Regulations (2005) include in their stated purpose (Article 2) a focus on preventing the international spread of disease “…in ways that are commensurate with and restricted to public health risks, and which avoid unnecessary interference with international traffic and trade” [24]. It is therefore important to build on the initial work presented here to further refine the circumstances and types of countries where the level of public health risk, and potential health gain, might justify border closures and indicate when they should be reopened.

## Supporting information

S1 Appendix Model in excel. (XLSX)

S2 Appendix Additional results. (DOCX)
