# Supplementary material for: Protecting an island nation from extreme pandemic threats: Proof-of-concept around border closure as an intervention
Source: PLoS One. 2017 Jun 16;12(6):e0178732. doi: 10.1371/journal.pone.0178732 (PMC5473559; doi:10.1371/journal.pone.0178732)
Supplement: S2 Appendix — (DOCX) [file pone.0178732.s002.docx]

**S2 Appendix: Additional Results**

**Additional results**

Figure A. Threshold analysis indicating the proportion of the New Zealand population dying in a pandemic (x-axis), vs number of weeks the border must be closed to forgo an equivalent amount of international visitor revenue. That is, there is net societal benefit (it is cost-beneficial) to close borders for 50 weeks if 0.15% of the population will otherwise die from the pandemic. Scenario A (0.275% population mortality) indicated by the circle.

**
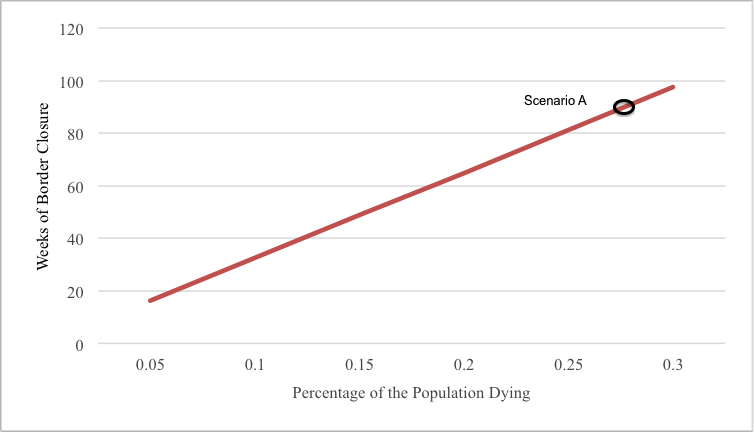
**

Figure B. Tornado plot showing the impact on net economic benefit of varying key parameters in Scenario A (see Table 2, sub-scenario 1, 26 weeks border closure). It remains cost-beneficial to close borders when these parameters are set to 2.5 and 97.5^th^ centiles

**
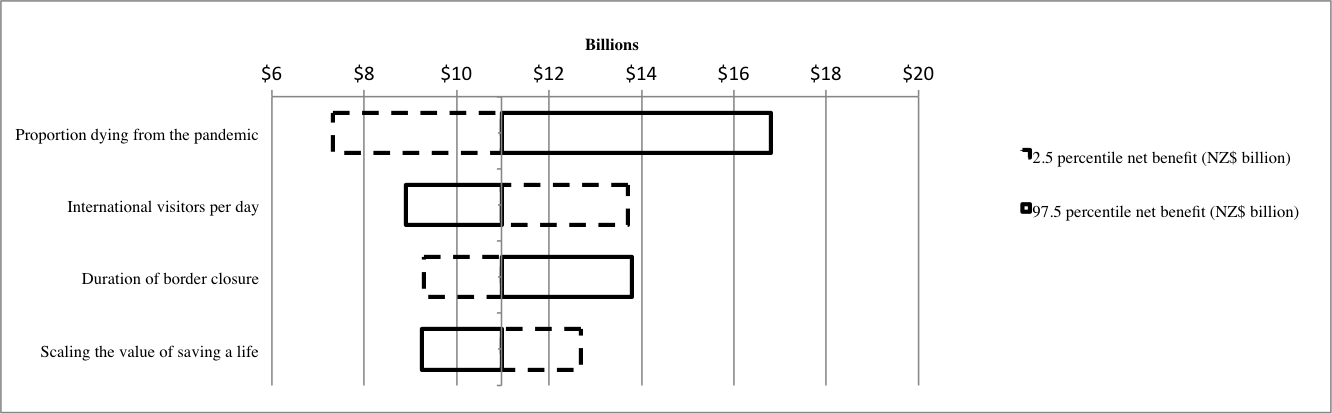
**

Figure C. Tornado plot showing the impact on net economic benefit of varying key parameters in Scenario A, when QALYs gained are not monetized

**
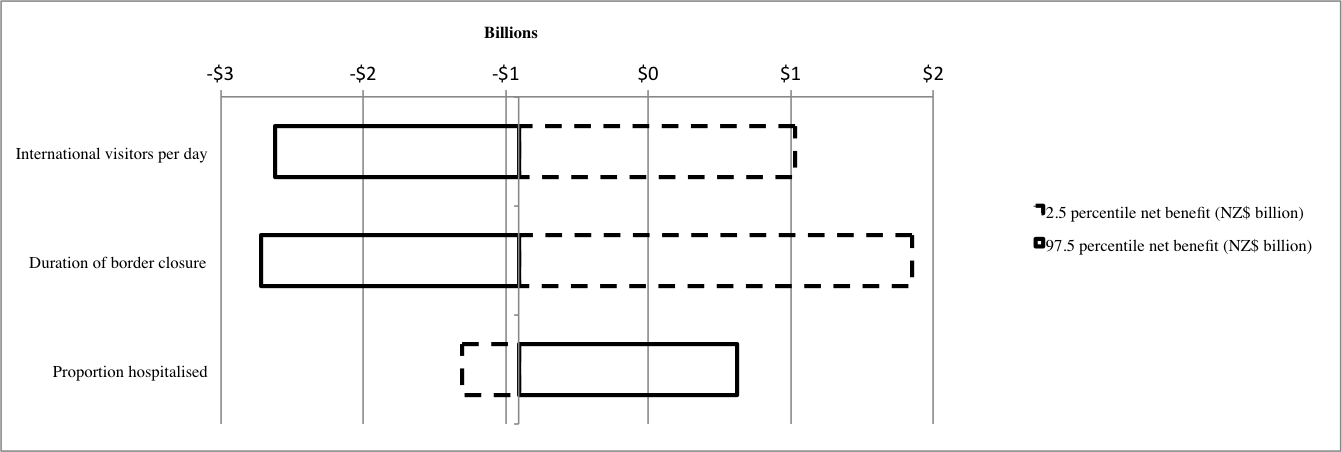
**
